# Applying the Behavioural Science Approach to Realist Reviews and Evaluations (BARR/E): A Protocol for a Review of Antidepressant Deprescribing Interventions in Primary Care

**DOI:** 10.3390/bs16071251

**Published:** 2026-07-22

**Authors:** Bethany Atkins, Caroline Smith, Debi Bhattacharya, Sion Scott

**Affiliations:** School of Health Sciences, University of East Anglia, Norwich NR4 7TJ, UK; bethany.atkins@uea.ac.uk (B.A.); caroline.smith@uea.ac.uk (C.S.); d.bhattacharya@uea.ac.uk (D.B.)

**Keywords:** psychotropic, mental health, depression, anxiety, obsessive compulsive disorder, post-traumatic stress disorder, determinants, general practice

## Abstract

1-in-5 people are prescribed an antidepressant for a common mental disorder (CMD), such as depression or anxiety. Most people do not require lifelong treatment, however more than half continue antidepressants for ≥2 years despite no ongoing symptoms. Antidepressant deprescribing is not routine practice in primary care and healthcare professionals report barriers including concerns about withdrawal effects, perceived patient attachment to antidepressants and limited availability of withdrawal-informed support. Drawing on realist principles and behavioural science, this review will identify behavioural mechanisms and contexts from academic and grey literature to formulate programme theories describing how to facilitate primary care healthcare professionals to deprescribe antidepressants. The review is guided by the Behavioural science Approach to Realist Reviews/Evaluations (BARR/E) and will follow five steps: (1) define the review scope, (2) develop initial programme theories, (3) evidence search, (4) selection and appraisal and (5) data extraction and synthesis. Peer-reviewed literature will be identified through MEDLINE, Embase, PsycINFO and CINAHL, supplemented by grey literature identified through expert consultation, database and citation searches. The evidence will be assessed for relevance, rigour and richness, and data extracted and synthesised to develop context–mechanism–outcome configurations. Findings will be reported in accordance with RAMESES quality and publication standards.

## 1. Introduction

1-in-6 people in England have a common mental disorder (CMD) such as depression and anxiety (NHS England Digital, 2016). Non-pharmacological interventions such as cognitive behavioural therapy (CBT) are recommended first-line treatments (NICE, 2022; Royal College of Psychiatrists, 2019). However, 1-in-5 of the population (9 million adults) are prescribed an antidepressant for a CMD with rates doubling over the past 17 years (McCarthy, 2013).

Antidepressants provide symptom relief during an acute life event (Pigott et al., 2010). The Royal College of Psychiatrists recommends that most people do not need antidepressants for more than 6–12 months following symptom resolution (Royal College of Psychiatrists, 2019). Unfortunately, antidepressant deprescribing is not routine practice in primary care and over half of people prescribed an antidepressant have this continued for ≥2 years despite no longer having symptoms of a CMD (Johnson et al., 2012). This is despite the incidence of side effects, likelihood of them becoming irreversible, and the risk of withdrawal effects all increasing with duration of antidepressant treatment (Bet et al., 2013; Davies & Read, 2019).

Between 60–75% of people report experiencing at least one side effect from their antidepressant (Bet et al., 2013; Campos et al., 2021). Whilst some side effects are mild and cease upon discontinuation of the antidepressant, others impose significant burden on quality of life. Excessive daytime sleepiness, hyperhidrosis and emotional blunting affect greater than 1 in 10 people taking antidepressants (Bet et al., 2013; Campos et al., 2021). Prolonged use of antidepressants increases the risk of side-effects becoming irreversible; for example, up to 80% of people taking antidepressants develop sexual dysfunction and 10–34% of these cases do not resolve with antidepressant discontinuation (Balon, 2006; Ben-Sheetrit et al., 2023). Furthermore, a 2019 systematic review highlights that abrupt discontinuation of antidepressants is frequently associated with withdrawal effects, reported by 56% of people and lasting between a few weeks to several months (Davies & Read, 2019). These effects include flu-like symptoms, sensory disturbances and sleep disruption (Davies & Read, 2019). Some describe these as mild, however, 46% of people experience severe withdrawal effects (Davies & Read, 2019).

Over half of people taking antidepressants for a CMD report not wanting to take it anymore and 92% would stop if deprescribing was recommended by their healthcare professional (HCP) (Cartwright et al., 2016; Lundby et al., 2024). A survey of (n = 180) people who had taken antidepressants for 3–15 years indicates that insufficient support from primary care HCPs disempowers patients to act on feelings that their antidepressant may no longer be needed (Cartwright et al., 2016). Conversely, when HCPs are proactive towards antidepressant deprescribing, patients interviewed in a Dutch primary care antidepressant deprescribing randomised controlled trial indicated that any “fear to discontinue would diminish” (Eveleigh et al., 2019). There is therefore a need for HCPs to work with patients to deprescribe antidepressants for CMDs when appropriate.

Safely and effectively deprescribing antidepressants is a complex behaviour that involves HCPs reviewing appropriateness, initiating deprescribing discussions with patients, referring/signposting them to locally available non-pharmacological support and monitoring for CMD relapse. Our scoping review identified several determinants to HCPs deprescribing antidepressants, including a lack of motivation due to perceiving antidepressant deprescribing as a low priority for primary care, fear of the professional consequences of CMD relapse and withdrawal effects, and not knowing where to refer patients to for non-pharmacological support (B. Atkins et al., 2025).

Our 2025 scoping review identified eight studies reporting interventions to facilitate HCPs to deprescribe antidepressants (Bowers et al., 2021; Eveleigh et al., 2018; Huijbers et al., 2023; Johnson et al., 2012; Kendrick et al., 2020; Muskens et al., 2013; Wallis et al., 2023; Wentink et al., 2019). These existing interventions primarily focus on providing training, education and information for HCPs regarding how to discontinue antidepressants, and some provide access to non-pharmacological alternatives to antidepressants for patients. Whilst these innovations demonstrate pockets of activity to support antidepressant deprescribing, their evaluation and reporting is varied, thus there is no clear ‘best’ model for addressing antidepressant overprescribing. These innovations, however, offer significant learning.

There is a need to draw together learning from academic and grey literature to establish what components of antidepressant deprescribing interventions work, for whom and in what circumstances to achieve the desired effects. Drawing on realist principles and behavioural science, we will identify behavioural mechanisms and contexts from academic and grey literature to formulate programme theories (PTs) describing how to facilitate primary care HCPs to deprescribe antidepressants.

### 1.1. Realist Methodology

A realist review is similar to the traditional systematic review in that it synthesises evidence from diverse sources, including academic and grey literature (Pawson et al., 2005). However, unlike traditional empirical approaches that focus on determining whether an intervention is effective, a realist review is theory-driven and designed to answer the question: “What works, for whom, in what circumstances, how, and why?” (Jagosh, 2019). It does this by examining and explaining the underlying mechanism(s) through which complex interventions work, or do not work, while considering the contextual conditions, settings and circumstances that surround an intervention and shape its outcomes (Pawson et al., 2005).

A realist review examines sets of Context–Mechanism–Outcome configurations (CMOcs). Context is typically defined as the background and setting surrounding an intervention that may play an integral part in producing its outcome(s). For example, institutional settings, economic and organisational structures, political conditions and cultural norms may trigger or inhibit the mechanisms within an intervention (De Weger et al., 2020; Greenhalgh & Manzano, 2021). Definitions of mechanism vary within the realist community, but it is typically understood as the ‘hidden’ process that link contexts and outcomes. Mechanisms are the interactions between an intervention’s components and peoples’ reactions to those components, or as the explanations for how and why outcomes occur as a result of an intervention (Dalkin et al., 2015). Mechanisms help explain what is triggered or inhibited in people because of an intervention and why an intervention may succeed in one setting and fail in another. This is because a mechanism is only triggered in the right contextual conditions. Outcomes are the (un)intended and (un)expected changes that occur following the introduction of an intervention (De Weger et al., 2020). Changing the intervention Context can trigger or inhibit an intervention’s underpinning Mechanism, which consequently changes the Outcomes (Pearsons et al., 2023).

Realist methodology uses ‘programme theories’ to understand and represent the causal forces underpinning the success or failure of interventions (Wong et al., 2013). Initial Programme Theories (IPTs) are developed to hypothesise how and why an intervention may or may not work. They are often presented as ‘if/then’ statements; if a particular context is present, then a specific mechanism is triggered/inhibited, leading to a particular outcome. They therefore propose the theoretical relationships between contextual conditions, the mechanisms they trigger/inhibit, and the outcomes that follow. The IPTs are then tested against the evidence included in the review to determine whether the hypotheses are supported, refined, or refuted (Pawson et al., 2005). This testing produces final programme theories which are grounded in the synthesised evidence and presented as CMOcs.

### 1.2. Applying Behavioural Science to Realist Approaches

In a traditional realist review, there is boundary ambiguity between reviewers when defining and identifying what is a context and what is a mechanism (Shaw et al., 2018). This is because the boundary between contexts and mechanisms can often be conceptually blurry and thus different researchers may classify the same element differently. The application of an a priori theory, in this case a behavioural science theory/framework, in the development of programme theories overcomes this challenge as it offers recognised definitions that can support uniformity in the interpretation and reporting of context and mechanism data (Bhattacharya et al., 2022; Kantilal et al., 2022). The application of behavioural-science-underpinned realist research is an emerging approach and is termed the Behavioural science Approach to Realist Reviews/Evaluations (BARR/E) (Bhattacharya et al., 2022; Kantilal et al., 2022; Smith et al., 2023, 2024). This approach has been used in realist reviews of interventions to support HCPs to provide cancer self-management support (Kantilal et al., 2022),opioid-tapering interventions (Bhattacharya et al., 2022) and oropharyngeal dysphagia screening (Smith et al., 2024).

In this review, mechanisms will be conceptualised as behavioural mechanisms which are ‘the process by which the active ingredients of an intervention affect behaviour’ (Michie et al., 2018). In many cases there may well be a causal pathway of behavioural mechanisms, e.g., addressing a knowledge gap may then lead to building an individual’s confidence. In this example, the most proximal mechanism is knowledge and the ‘downstream’ mechanism is confidence. Thus, delivering confidence building activities without first addressing the knowledge gap will not achieve the desired outcome. Using the BARR/E, the most proximal mechanism is hypothesised in an IPT.

**Figure 1:**
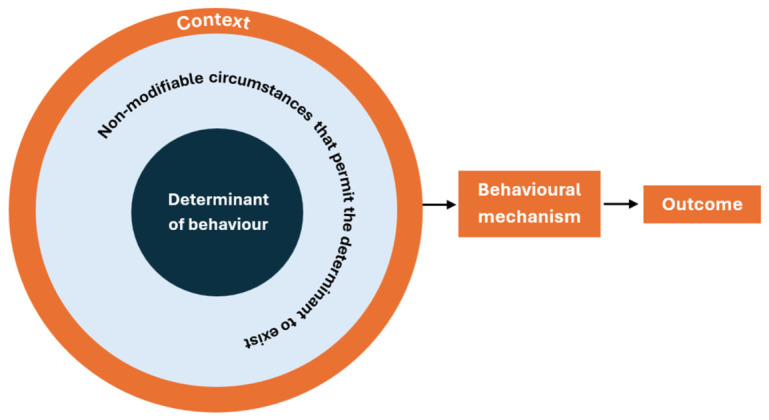
Context, mechanism and outcome configuration in the Behavioural science Approach to Realist Reviews/Evaluations (BARR/E).

Context comprises two elements: a determinant of the target behaviour and the non-modifiable circumstances that permit the determinant to exist (Bhattacharya et al., 2022; Kantilal et al., 2022). Representing context in this way is key as it anchors each IPT in realist logic by specifying the enabling or constraining conditions that permit the target behaviour to be undertaken. Figure 1 provides an overview of the context, mechanism and outcome configuration in the Behavioural science Approach to Realist Reviews/Evaluations (BARR/E).

Using the example of the target behaviour being someone regularly going for a run, one might develop a hypothesis that having a pavement is an enabler of going for a run that is only triggered in the non-modifiable context of being in a city, because people believe that it is safer to run on the pavement than a road with busy traffic. The most proximal mechanism in this example is beliefs about consequences. Outside of a city context, having a pavement is no longer an enabler of going for a run because people feel safe to run on quiet country roads. This IPT presented as a CMO configuration is:

*In the city (C^N-M^), pavements facilitate people to run (C^D^) because people believe that it is safer to run on the pavement than a road with busy traffic (M), leading to people regularly going for a run (O)*

### 1.3. Aim and Objectives

The aim of this realist review is to synthesise learning from published and grey literature to understand what works, for whom, under what circumstances and how, to facilitate primary care HCPs to safely and effectively deprescribe antidepressants.

The objectives of this review are to:

- Identify academic and grey literature relevant to antidepressant deprescribing in the primary care setting
- In collaboration with key stakeholders, develop and prioritise IPTs for testing against the academic and grey literature
- Develop Programme Theories (PTs) describing how interventions facilitate primary care HCPs to deprescribe antidepressants

## 2. Materials and Methods

This review protocol has been registered with the Prospective Register of Systematic Reviews (PROSPERO, n.d.). We will report the study results according to the ‘Realist And Meta-narrative Evidence Syntheses: Evolving Standards’ (RAMESES) quality and publication standards (Wong et al., 2013, 2014).

Throughout the review, we will work with our Expert by Profession (EBP) stakeholder group and Patient and Public Involvement (PPI) members. Our EBP group comprises nine members who have been purposefully sampled to reflect diverse professional backgrounds and system perspectives, including mental health charity directors, HCPs (psychologists, psychiatrists, mental health nurses, social prescribers and community mental health pharmacists) and senior health system commissioners and policy makers. Our PPI group comprises seven members with lived experience of taking an antidepressant and who have a mix of positive, negative or no experience of deprescribing

This review is guided by the BARR/E methodological approach (Bhattacharya et al., 2022; Kantilal et al., 2022; Smith et al., 2023, 2024) and will follow the five steps adapted from Pawson et al. for conducting a realist review (Pawson et al., 2005): (1) define the review scope, (2) develop initial programme theories, (3) search for evidence, (4) select and appraise evidence, and (5) extract and synthesise data. While these steps are presented sequentially below, the conduct of a realist review is iterative; in practice, steps may overlap or progress concurrently. For example, we may conduct additional searches after data extraction and synthesis in response to the evidence and the refinement of programme theories.

**Step 1. Define the review scope**

Preliminary research questions have been developed using the expertise and experience of the core research team, including pharmacists, a physician, a mental health nurse, a realist methodologist and a behavioural scientist. The preliminary research questions guiding this review are:

1. What are the contextual determinants of primary care HCPs deprescribing antidepressants?
2. What are the behavioural mechanisms by which interventions to facilitate primary care HCPs to deprescribe antidepressants result in their outcomes?
3. How do contextual determinants influence the behavioural mechanisms?

Questions may be refined in response to feedback from our PPI members and new learning in line with the iterative nature of realist methodology (Pawson et al., 2005).

**Step 2. Initial Programme Theory (IPT) development and prioritisation**

Initial Programme Theories will initially be inductively generated by the research team (BA, SS, DB) using experiences and knowledge from a related antidepressant deprescribing scoping review (B. Atkins et al., 2025). The inductively generated IPTs will then be mapped to the relevant Theoretical Domains Framework (TDF) domains based on the behavioural mechanism of action. The TDF is a synthesis of 33 behaviour change theories for understanding and developing strategies to change behaviour organised into 14 theoretical domains (L. Atkins et al., 2017). The 14 TDF domains are linked to a taxonomy of Behaviour Change Techniques (BCTs), which are strategies to change behaviour. The TDF provides a structured, yet flexible, approach to building programme theories that will allow us to identify a broad range of influences on HCP behaviour that may not be clear from the evidence alone, as well as explore the influence of context at different levels such as individual, team, organisational and system.

To consider a broader range of influences on HCP behaviour, additional IPTs will be deductively generated using behavioural mechanisms related to TDF domains which have no inductive IPTs mapped. Deductive development of IPTs will continue to be guided by the scoping review and the research team’s experiences; they are not intended to be exhaustive at this stage (Bhattacharya et al., 2022; Kantilal et al., 2022).

### 2.1. Online Survey

The IPTs will be developed into an online survey hosted on MS Forms. The survey will invite the EBP stakeholder group to indicate which ones they perceive important for facilitating primary care HCPs to deprescribe antidepressants. We will also invite respondents to provide comments on the IPTs and propose additional IPTs for discussion in the subsequent stakeholder meeting.

We will calculate the percentage of stakeholders indicating that an IPT should be prioritised for testing using the following a priori criteria (Diamond et al., 2014):

- If 100% of stakeholders agree that the IPT should be prioritised, it will be categorised as ‘progress to testing’
- If 60–99% of stakeholders agree that the IPT should be prioritised, it will be categorised as ‘for discussion’
- If less than 60% of stakeholders agree that an IPT is important, it will be categorised as ‘discarded’

The progression of IPTs for testing will be treated as provisional until ratified by EBP members.

### 2.2. Stakeholder Meeting

We will present survey results in a 90-min online meeting with the EBP stakeholder group. We will present all categorised IPTs and facilitate the group to reach consensus regarding their prioritisation of IPTs for testing. Following RAMESES guidance (Wong et al., 2014), all ‘discarded’ IPTs will be archived but re-introduced if there is evidence to support them (see Data extraction and synthesis).

**Step 3. Evidence Searches**

We will search peer-reviewed literature in the following databases: MEDLINE, Embase, PsycINFO and Cumulative Index to Nursing and Allied Health Literature (CINAHL). These databases provide strong complementary coverage of health, nursing, allied health and behavioural science research (Bramer et al., 2017). We will refine and apply the search terms used in our published scoping review (B. Atkins et al., 2025); these were developed in collaboration with an information specialist and will be refined in this study based on findings and with input from the EBP group. Supplementary File S1 provides the draft Medline search strategy. We will combine this with keyword and citation searches on Google Scholar and review of reference lists of eligible sources of published evidence.

We will conduct grey literature searches to identify evidence that is not published in academic journals, e.g., guidelines, policy reports, service evaluations, conference proceedings and theses. We will do this by:

- Searching grey literature databases such as The King’s Fund Library Database *
- Contacting authors or experts of antidepressant deprescribing interventions
- Scanning reference lists of articles included in the review (backward citation searching)
- Searching for studies that cite the articles included in the review (“snowballing” or forward citation searching)
- Searching trial or study registers

We will seek input from our PPI/EBP groups and core research team members to identify relevant publications or grey literature.

No date or geographic restrictions will be imposed on our initial search.

* Owing to the often limited search functionality of grey literature databases, we will search core concept terms (e.g., antidepressants, deprescribing, common mental disorder) individually and screen all retrieved evidence.

### 2.3. Inclusion and Exclusion Criteria

Evidence inclusion and exclusion criteria are presented in Table 1.

**Step 4. Selection and Appraisal of Evidence**

The search results will be downloaded into Zotero for de-duplication and then imported into Covidence to facilitate screening and appraisal.

To ensure accuracy and alleviate risk of bias, two reviewers (BA & SS) will independently review titles and abstracts of articles for full-text review against the inclusion and exclusion criteria (Aromataris et al., 2024; Waffenschmidt et al., 2019). If a study meets the eligibility criteria, or if a decision cannot be made based on the title and abstract alone, the full-text article will be retrieved.

All full-text articles will be screened against the inclusion and exclusion criteria and appraised according to their relevance (whether the content of the evidence can contribute to the testing of IPTs (Dada et al., 2023; Pawson et al., 2005)), rigour (whether the contents of the evidence have sufficient substance to make credible contributions to the testing of IPTs (Dada et al., 2023; Pawson et al., 2005)) and richness (the extent to which the evidence can contribute to the development of final PTs (Booth et al., 2013; Dada et al., 2023; Jagosh et al., 2011)). Relevance of evidence will be assessed to distinguish between conceptually rich and weaker evidence for providing explanations for PT development. The following will be used to determine the rigour of included evidence:

- The Mixed Methods Appraisal Tool, to evaluate primary quantitative and qualitative studies (Hong et al., 2018)
- The Authority, Accuracy, Coverage, Objectivity, Date, Significance checklist for grey literature (Tyndall, 2010)

All evidence will be screened and appraised at full-text by two reviewers (BA & SS), with discrepancies to be discussed and resolved with a third reviewer (DB).

**Step 5. Data Extraction and Synthesis**

Evidence included after full-text screening and appraisal will be imported into a bespoke data extraction tool in MS Excel for data extraction and synthesis.

Evidence will first be coded by two reviewers (BA & SS) relating to context, mechanism and outcome. Next, the coded data will be mapped to the corresponding TDF domain and IPT, or where a relevant IPT does not exist, used to create a new IPT. Data will be organised and labelled to make clear where the coded data confirms or contradicts an IPT. If there is evidence to support any ‘discarded’ IPTs, these will be re-introduced. Following RAMESES guidance (Wong et al., 2014), we will make explicit the evidence underpinning all IPTs as they are reinstated, refined, or combined.

Following data synthesis, the research team will convene a second 90-min stakeholder meeting with the EBP group to discuss the prioritised IPTs and their development into the proposed final PTs, presented as CMOcs. These will be contextualised for use in the UK primary care context in a subsequent study.

## 3. Discussion

This behavioural science realist review will develop, test and refine programme theories explaining what components of antidepressant deprescribing interventions work, for whom and in what circumstances to achieve the desired effects. This understanding will permit the development of antidepressant deprescribing interventions that afford flexibility for adaptation according to contextual factors such as local resource and infrastructure.

The application of the Behavioural science Approach to Realist Reviews/Evaluations (BARR/E) (Bhattacharya et al., 2022; Kantilal et al., 2022; Smith et al., 2023, 2024) permits the integration an a priori behavioural science framework. This approach provides a shared theoretical language that supports uniformity in identifying and reporting contexts and mechanisms. By developing PTs using the ‘recognised’ behavioural mechanisms within the TDF, and through the TDFs linkage to behaviour change techniques (BCTs), which are the active ingredients of behaviour change interventions, the realist review will provide a framework from which to develop an intervention to facilitate primary care HCPs to deprescribe antidepressants.

The findings from this review will be contextualised to the UK setting in a subsequent realist evaluation and used to co-design an intervention to facilitate primary care HCPs to deprescribe antidepressants.

## Figures and Tables

**Table 1:** Inclusion and exclusion criteria according to Population, Concept and Context.

	Inclusion Criteria	Exclusion Criteria
Population	Healthcare professionals	Individuals who are not healthcare professionals
Context	Primary care setting (inclusive of nursing/care homes); focus on the discontinuation of antidepressants prescribed for mild-moderate depression, generalised anxiety disorder (GAD), obsessive compulsive disorder (OCD), post-traumatic stress disorder (PTSD), panic disorder and/or social anxiety disorder.	Focus on the discontinuation of antidepressants prescribed for non-mental health conditions (e.g., chronic pain)
Concept	Reporting barriers and/or enablers to HCPs discontinuing antidepressants, and/or interventions to facilitate HCPs to support patients through the discontinuation process.	N/A
Publication	Original peer reviewed research (protocols, randomised controlled trial (RCT), quasi-experimental, cohort study, qualitative and case studies); published in the English language. Grey literature (e.g., guidelines, policy reports, service evaluations, conference proceedings and theses).	Conference abstracts, systematic reviews *, editorials, reviews, case reports, and non-research letters.

* Systematic reviews are ineligible but if retrieved by the search will be reviewed for the purpose of finding additional relevant studies (through scanning reference lists).

## Data Availability

The original contributions presented in this study are included in the article. Further inquiries can be directed to the corresponding author.

## Supplementary Materials

The following supporting information can be downloaded at: https://www.mdpi.com/article/10.3390/bs16071251/s1, File S1: Search strategy.

## Abbreviations

The following abbreviations are used in this manuscript:

BCT	Behaviour Change Technique
CMOcs	Context-Mechanism-Outcome configurations
CINAHL	Cumulative Index to Nursing and Allied Health Literature
HCP	Healthcare professional
TDF	Theoretical Domains Framework
ICB	Integrated Care Board
ICS	Integrated Care System
IPT	Initial Programme Theory
PT	Programme Theory
PPI	Public and Patient Involvement
EBP	Expert by Profession
MoA	Mechanism of Action
RAMESES	Realist And Meta-narrative Evidence Syntheses: Evolving Standards
