# Supplementary material for: Applying the Behavioural Science Approach to Realist Reviews and Evaluations (BARR/E): A Protocol for a Review of Antidepressant Deprescribing Interventions in Primary Care
Source: Behav Sci (Basel). 2026 Jul 22;16(7):1251. doi: 10.3390/bs16071251 (PMC13404939; doi:10.3390/bs16071251)
Supplement: Supplementary file 1 [file behavsci-16-01251-s001.zip › behavsci-4374590-supplementary.pdf]

Supplementary File S1. Example search strategy for Ovid MEDLINE

1	exp Depression/
2	(depression or depressive or depressed).ti,ab,kw,sh.
3	exp Anxiety Disorders/
4	generali?ed anxiety disorder*.ti,ab,kw,sh.
5	GAD.ti,ab,kw,sh.
6	exp Obsessive-Compulsive Disorder/
7	obsessive compulsive disorder*.ti,ab,kw,sh.
8	OCD.ti,ab,kw,sh.
9	exp Stress Disorders, Post-Traumatic/
10	post-traumatic stress disorder*.ti,ab,kw,sh.
11	PTSD.ti,ab,kw,sh.
12	exp Panic Disorder/
13	panic disorder*.ti,ab,kw,sh.
14	exp Phobia, Social/
15	social anxiety disorder*.ti,ab,kw,sh.
16	social phobia*.ti,ab,kw,sh.
17	or/1-16
18	exp Antidepressive Agents/
19	antidepress*.ti,ab,kw,sh.
20	exp Selective Serotonin Reuptake Inhibitors/
21	selective serotonin re-uptake inhibitor*.ti,ab,kw,sh.
22	exp Sertraline/
23	exp Fluoxetine/
24	exp Citalopram/
25	(Sertraline or Fluoxetine or Citalopram).ti,ab,kw,sh.
26	exp Monoamine Oxidase Inhibitors/
27	Monoamine Oxidase Inhibitor*.ti,ab,kw,sh.
28	exp Tranylcypromine/
29	exp Phenelzine/
30	exp Isocarboxazid/
31	(Tranylcypromine or Phenelzine or Isocarboxazid).ti,ab,kw,sh.
32	exp Antidepressive Agents, Tricyclic/
33	tricyclic antidepressant*.ti,ab,kw,sh.
34	exp Amitriptyline/
35	Amitriptyline.ti,ab,kw,sh.
36	exp Dothiepin/
37	Dothiepin.ti,ab,kw,sh.
38	dosulepin.ti,ab,kw,sh.
39	exp Doxepin/
40	doxepin.ti,ab,kw,sh.
41	exp "Serotonin and Noradrenaline Reuptake Inhibitors"/
42	"serotonin and noradrenaline reuptake inhibitor*".ti,ab,kw,sh.
43	exp Duloxetine Hydrochloride/
44	duloxetine.ti,ab,kw,sh.
45	exp Venlafaxine Hydrochloride/
46	venlafaxine.ti,ab,kw,sh.

47 or/18-46
48 17 and 47
49 exp Deprescriptions/
50 (taper* or wean* or detox* or withdraw* or discontinu* or cease or cessation or terminat* or remove* or stop* or substitut* or deprescri*).ti,ab,kw,sh.
51 ((dose or dosage or medicine* or medication or antidepressant*) adj1 (reduc* or consumption or lower* or decreas*)).ti,ab,kw,sh.
52 (prescrib* adj3 (reduc* or lower* or stop*)).ti,ab,kw,sh.
53 49 or 50 or 51 or 52
54 48 and 53
55 barrier*.ti,ab,kw,sh.
56 facilitator*.ti,ab,kw,sh.
57 enable*.ti,ab,kw,sh.
58 interven*.ti,ab,kw,sh.
59 train*.ti,ab,kw,sh.
60 educat*.ti,ab,kw,sh.
61 implement*.ti,ab,kw,sh.
62 service.ti,ab,kw,sh.
63 program*.ti,ab,kw,sh.
64 strateg*.ti,ab,kw,sh.
65 or/55-64
66 exp General Practice/
67 general practi*.ti,ab,kw,sh.
68 exp Primary Health Care/
69 primary health care.ti,ab,kw,sh.
70 primary care.ti,ab,kw,sh.
71 exp Family Practice/
72 family practice.ti,ab,kw,sh.
73 primary medical care.ti,ab,kw,sh.
74 or/66-73
75 65 and 74
76 54 and 75
77 limit 76 to english language
78 exp Animals/
79 exp Humans/
80 78 not 79
81 77 not 80
82 remove duplicates from 81
